# An *in vitro* wear behavior analysis of polymer composite for biomedical application

**DOI:** 10.6026/973206300200950

**Published:** 2024-08-31

**Authors:** Arul Jeyakumar Arputham, Queen Alice Arul, Padmapriya Mahalingam, Dipanjan Debnath

**Affiliations:** 1 Department of Mechanical Engineering, SRM University, Chennai, Tamil Nadu, India; 2 Department of Dentistry, AIIMS (All India Institute of Medical Sciences) Kalyani, West Bengal, India; 3 Department of Conservative Dentistry & Endodontics, Government Dental College, Chennai, Tamil Nadu, India

**Keywords:** PEEK composite, Basalt fiber, Wear resistance, Coefficient of Friction, Implant material

## Abstract

The tribological performance of basalt fiber reinforced PEEK material especially used as a biomaterial in many biomedical and dental applications is of interest. The specimens of three different weight fractions of PEEK and basalt fiber are fabricated as per ASTM G99 standards. The prepared specimens are having PEEK and basalt fiber in the weight percentage of 90:10, 80:20 and 70:30 ratio and named as PBC 1, PBC 2 and PBC 3 respectively. The specimens are subjected to pin-on-disc test using EN31 steel as the sliding disc material. The hardness of PBC 2 specimen shows a better value of 50.74 HRB. Wear resistance is comparatively less when Basalt weight percentage increases from 10% to 20%, but further increase of basalt fiber in the composite, the wear resistance drops down. Similarly, the COF values also noted high for PBC 2 compared to pure PEEK, PBC 1 and PBC 3 composites. PBC 2 sample is found to be better with high wear resistance.

## Background:

Titanium based alloys are used as dental implants till the end of 1960 [1]. Ti-alloys exhibits good physical, chemical, mechanical and biocompatibility properties [2]. However, Ti-alloys have an excellent modulus will create stress-shielding effect which results in implant failure [3]. Also, Titanium implants are subjected to clinical problems, like rare metal allergic reaction, surface wear and infection due to peri-implantitis [4]. A novel material to substitute Titanium implants, with zirconia [5] and polymeric compounds, such as polyether ether ketone (PEEK) have arrived [6]. The essential requirement of a material to understand its tribological properties are high contact temperature, chemical resistance, wears resistance and better mechanical behaviors [7]. PEEK was first synthesized by Bonner, which is a thermoplastic polymer with high heat resistance and good mechanical strength [6]. PEEK is an aromatic molecule with ketone and ether functional groups added to the ring structure, which is basically a poly aryl ether ketone family [8]. PEEK has a strong bonding aromatic ring structure capable of holding any number of ethers along with firm carbonyl group [9]. PEEK shows a stable configuration even in sterilization process due its high melting point and wears resistance [10,11]. Other than PEEK used as medical Implants, it is commercially used in aircraft and turbine blades [12, 13-14]. In case of orthopedic application PEEK plays a vital role in replacing metal implants in human body [15]. The annealing process increase the mechanical properties of the composites such as toughness, wear resistance and fatigue strength [16, 17, 18-19]. PEEK implants are prepared by hot compression molding process to improve high corrosive resistance and improved tribological properties by refining the grains in the composites [20]. Various types of reinforcement such as natural fiber and organic fiber are used as long and short fibers to improve the wear resistance, to enhance the stiffness and cost [21, 22]. In general, 20 wt% of fiber is reinforcement in PEEK matrix to improve the wear behavior of the composites [23-24]. In some composite metal particles are added as fillers in the matrix to improve the strength of the composites. Basalt fibers are used as reinforcement in medical devices since they are more bio compatible and are not harmful to human body [25]. PEEK with carbon filler shows an improved tensile strength at critical Environment [26]. The PEEK also shows a better glass transition of 143°C.

Therefore, it is of interest to report about the different pattern of wear property behavior of three different weight proportions of basalt natural fiber reinforced PEEK polymer composite.

## Materials and Methods:

## Materials:

PEEK is purchased from Sri Krishna Polymers, Chennai, India. Saline treated basalt short fiber of 2 to 3 mm length with diameter 13 µm and melting point of 1800°K is procured from Muktagiri private limited, Mumbai, India. Basalt fiber is the reinforcement and Poly Ether- Ether Ketone (PEEK) is the matrix material, as shown in Figure 1(see PDF).

## Preparation of composites:

PEEK and fiber are mixed in three different volume fractions of 90/10, 80/20 and 70/30 and the homogenous mixing of PEEK and basalt fiber is carried out hand mixture operating speed of 350 rpm for a period of 2 min. The localized agglomeration of PEEK matrix along with the basalt fiber occurs due to the silane treatment of basalt fiber [27]. The volume fractions of 100/0, 90/10, 80/20 and 70/30 are named as Pure PEEK, PBC 1, PBC 2 and PBC 3 respectively. The mixture is dried for 2 h at 80°C to remove moisture content in a hot oven.

## Compounding of composites:

Hot compression molding is used in fabrication of thermoplastic polymer composite. Hot compression mold (HALD420) with a maximum compression load capacity of 100 tons and operating temperature of 500°C is employed for fabrication of the composites. The process begins with mixing the materials homogenously appropriate to their weight fractions. The loaded mold is then pre-heated to 380°C. The mold is transferred to the press where it is subjected to pressure of 35 bar.

The specimens are heated up to 380°C. The matrix melts under pressure and heat and blends with fiber to form composite. Hot compression molding does not require any post processing work, allowing the sample to cool by itself in the mold is well enough [28]. Compression specimens are shown in the Figure 2a,b,c(see PDF). The final specimens are hence obtained by hot compression molding. Three different samples, namely PBC 1 (PEEK 90%, BASALT 10%) PBC 2 (PEEK 80%, BASALT 20%) and PBC 3 (PEEK 70%, BASALT 30%) are fabricated using hot compaction molding.

## Hardness test:

Hardness of the composites was tested in Rockwell Hardness testing machine at 100 kgf and 12.70 mm ball indenter B Scale (SRMIST, KTR). The hardness values of the composites were checked to identify the disc material to be used in pin-on-disc apparatus. It is clear that the hardness of the disc should be maximum, compared to material that slide on it to understand the wear properties of the materials.

## Wear test:

The sliding wear resistance of all PEEK specimens PBC 1, PBC 2 and PBC 3 fabricated using the hot compression molding technique are investigated under similar conditions. The dry sliding wear test is carried out for sliding speeds from 1000 m to 4000 m and for different loads of 20 N and 40 N in a pin on disc apparatus as shown in Figure 3 (see PDF). Frictional and wear behavior for dry sliding condition of the cylindrical composites were made according to ASTM G99 test standard and examined using Pin-on-disc tester [29]. The emery sheet of grit size 320, 600, 1000 and 1200 were used to polish the sliding surface before each experiment to get the effective contact.

The test was carried at Normal Load and constant sliding velocity of 350 rpm for varying sliding distances of 1000m to 4000m. The wear loss based on applied load, reinforcement content and various sliding distance, COF and change in weight of the composite were recorded and analyzed. All prepared composites such as PBC1, PBC2 and PBC3 were subjected to wear test with trial of 5 specimens in each. An average of this is taken for the analysis.

## Result & Discussion:

The results obtained from the Rockwell hardness B scale tests are given in the Table 1. From Table 1, the average hardness values of PBC1, PBC2 and PBC3 are 49.53 HRB, 50.47 HRB and 50.23 HRB respectively. The Hardness value of pure PEEK material is 48.73 HRB, which is lesser than the hardness of the composite samples. It is noted that the hardness value of PBC2 is greater than the hardness values of the samples PBC1 and PBC3; This may be due to the moderate addition of basalt fiber (20 wt %) in the PEEK matrix (80 wt %) leads to an excellent bonding between the matrix and the fiber.

## Wear testing (pin-on-disc):

The pin on disc test results of pure PEEK, PBC 1, PBC 2 and PBC 3 composites are given in Table 2, Table 3, Table 4 and Table 5 respectively below.

## Co-efficient of Friction of Pure PEEK, PBC 1, PBC 2 and PBC 3 composites:

The COF of pure PEEK decreases as the sliding distance increases from 1000 m to 4000 m with constant velocity of 350 rpm for both loading conditions of 20 N and 40 N as indicated in the Table 2. The maximum COF for 20 N loads is 0.38 for a rotating distance of 1000 m. Similarly, a maximum COF is 0.45 is observed at 40 N loading conditions for 1000m sliding distance, as shown in Figure 4a (see PDF). The Figure 4b(see PDF) shows the COF with respect to sliding distance of PBC1 composite. The initial COF at 1000 m sliding distance under constant velocity of 350 rpm and normal load of 20 N is 0.3641. Further increase in the sliding distance from 2000 to 4000 m, the COF decreases to 0.2639 as indicated in Table 3. It is noted that the COF decreases as the sliding distance increases. Similarly, when the load is increased to 40N and with same 350 rpm speed the COF decreases from 0.4095 to 0.3094 for 1000m and 4000m sliding distance respectively. This may be due to the increase in sliding distance, may harden the contact surface and the asperities at the contact surfaces get fastened to the plastic zone and improves the hardness at the contact surfaces [30]. This may lead to increase in frictional force and hence the COF increases gradually with increase in the rotating distance.

The Figure 4c(see PDF) indicates the COF of the Composite PBC2, decreases from 0.3264 to 0.2394 as the sliding distance increases from 1000 meter to 4000 meter at constant sliding velocity of 350 rpm with 20N load. The frictional force increases as the load increases from 20N to 40N for 1000 meter sliding distance, the initial COF is 0.3564. Further increase in sliding distance to 4000 meter, the COF gradually decreases to 0.2991. The Table 4 showing the COF with respect to increase in sliding distance of PBC2. The maximum COF of PBC 2 is less compared to COF of PBC1 composites may be due to the fiber volume percentage increase in the composite, which may result in reduction in the frictional force. The Figure 4d (see PDF) reveals the COF decreases as the sliding distance increases in case of PBC 3 from 0.3241 to 0.2694 at a normal load of 20 N. When the load increases to 40 N the COF is 0.3771 after a sliding distance of 1000 m. The Table 5 shows the COF of PBC3 reduces to 0.3024 when the sliding length increases to 4000 m at 40N load and 350 rpm velocity of the disc.

## Wear Rate of PBC 1, PBC 2 and PBC 3 composites:

The Table 2 indicates wear behavior of pure PEEK material. The increase in wear rate from 145.63 X 10^-4^ m^2^/N to 152.74 X 10^-4^ m^2^/N as the sliding distance increases from 1000 m to 4000 m at 20 N load and 350 rpm. Similarly at 40 N load with same sliding velocity, the wear rate increases from 149.84 X 10^-4^ m^2^/N to 153.15 X 10^-4^ m^2^/N as the sliding distance increase from 1000m to 4000 m respectively as shown in Figure 5a (see PDF). This increase in wear is gradual which clearly indicates no substituting ingredients in the composite to reduce the frictional heat and lubricates the contacts to reduce the wear. The Table 3 indicates that the wear rate of PBC 1 composite with initial wear of 135.436 X 10^-4^ m^2^/N after the pin travel 1000 m of sliding, with a constant sliding velocity of 350 rpm and 20N load and further the wear rate increases to 142.048 X 10^-4^ m^2^/N as the sliding distance increases to 4000 m. Also, when the load is increased to 40N, the maximum wear rate is 142.429 X 10^-4^ m^2^/N at maximum sliding length of 4000 m as indicated in Figure 5b (see PDF). Figure 5a and Figure 5b (see PDF) indicates increase in the sliding length gradually increases the wear rate, whereas the maximum wear rate at maximum load in PBC 1 is 142.429 X 10^-4^ m^2^/N, which is more compared to PBC 2 of 130.943 X 10^-4^ m^2^/N (Table 4). This is due to increase in fiber content in the composite that reduces the material loss during sliding. The Figure 5c (see PDF) indicates in case of PBC 3 the wear loss increases with increase in sliding distance as in the case of PBC 1 & PBC 2. The Table 5 indicates the wear rate starts at 131.067 X 10^-4^ m^2^/N after taking 1000 m of sliding with 20 N load, whereas the wear loss increases to 137.466 X 10^-4^ m^2^/N, when the sliding distance increases to 4000 m at constant sliding velocity and same load. When the load increases to 40N, the wear rate after 1000 m of sliding distance is 134.856 X 10^-4^ m^2^/N. It increases to 137.835 X 10^-4^ m^2^/N as the sliding distance increases to 4000 m. In Figure 5d (see PDF) the average wear rate at 20N load for pure PEEK is 149.01 X 10^-4^ m^2^/N at 20N load and at 40N load the wear rate is 152 X 10^-4^ m^2^/N. The average wear rate of PBC 1 is around 138.01 X 10^-4^ m^2^/N which increases to 141.90 X 10^-4^ m^2^/N at 20N and 40N respectively. Whereas PBC 2 has less wear rate at 20N of 127.01 X 10^-4^ m^2^/N and at 40N the wear rate is 129.58 X 10^-4^ m^2^/N. Similarly, PBC 3 wear rate at 20 N is 133.94 X 10^-4^ m^2^/N and at increase load of 40 N the average wear rate is 136.28 X 10^-4^ m^2^/N. It is inferred that the wear rate is minimum in case of PBC 2 compared to PBC 1 and PBC 3. The Figure 5d(see PDF) indicates the wear rate under increase in load of all the composites. It is shown that wear loss for PBC2 is less compared to PBC1 which may be due to weak bonding in PBC 1 and PBC 3 under sliding load may cause agglomeration of PEEK matrix.

## Wear Loss based on Normal load of PBC 1, PBC 2 and PBC 3 composites:

The Table 2 indicates a gradual reduction in wear loss as the pure PEEK, slides over the contact surface with maximum wear loss of 0.0057gms; though the wear loss is minimum, the specimen needs adequate reinforcement to substantiate the frictional heat by which the wear resistance can be improved, compared to the other composites, wear loss is marginally high [31]. The wear loss is more in case of PBC 1 is shown in Figure 6a (see PDF) with increase in load and sliding distance. Whereas Figure 6b(see PDF) indicates less wear loss in PBC 2 when compared to PBC 1. In case of PBC 3 the wear loss increases as the normal load increases, but the increase in wear loss is less compared to PBC1 but more than PBC 2 composites as indicated in the Figure 6c (see PDF). The indication of blue shades represents less wear and the green color indicates the more wear loss in the contour plot. Loss due to wear is most pronounced when the load applied is increased beyond 20N to 40N and the sliding distance is increased 4000 meters. On a micro scale, characteristics of wear and friction of a material depend upon the growth, formation and disintegration of contact plateaus [32].

Figure 7a(see PDF) indicates the wear in terms of weight loss of the composites with respect to increase in basalt fiber reinforcement. The wear rate is more, when the reinforcement is 10 weight percentage of basalt fiber in the composite and when the normal load is high. Similarly, when reinforcement increases, the wear loss is less with respect to increase in the load for all the composites. The wear loss is less for PBC 2 compared to PBC 1 and PBC 3 with a maximum wear rate value of PBC 2 is 0.0014 g at 40 N and 4000 m sliding velocity. Whereas maximum wear of PBC 1 and PBC 3 at maximum load of 40 N are 0.0064 g and 0.003 g respectively. Figure 7b(see PDF) indicates the COF of the composites taken in this study. The maximum COF of 0.364 is observed at 10% basalt reinforced fiber at 40N load. Similarly, the COF decreases as the fiber loading in 20% in the composite and the load is 40N. From Figure 7a & Figure 7b (see PDF) it is shown, the increase in basalt content increases the wear resistance of the composite up to 20 weight percentage of basalt fiber and further increase in the basalt content of 30 weight percentage decreases the wear resistance [33].

## Conclusions:

The hardness value of 50.74 for PBC2 is higher among other samples. The increase of basalt fiber weight percentage from 10 % to 20 weight % in the composite improves the wear resistance, but further increase to 30 weight % marginally decreases the wear résistance. The wear loss increased as the normal load increases, from 20 to 40 N for all the three samples. This comparative study of wear characteristics of all three samples shows PBC2 to be a better wear-resistant composite that is found more reliable for dental and medical application and further future clinical studies have to be conducted.

## Funding:

This research did not receive any specific grant from any funding agencies.

## Disclosure statement:

All authors contributed equally in carrying out the research and in writing the manuscript and the authors report there are no competing interests to declare.

## Data availability statement:

The datasets generated during and/or analyzed during the current study are available from the corresponding author on reasonable request.

## Ethical Approval:

Ref. No: IEC/AIIMS/Kalyani/certificate/2024/245

## Figures and Tables

**Table 1:** Hardness of the sample in HRB

**Composites**	**Indentation 1**	**Indentation 2**	**Indentation 3**
Pure PEEK	48.3	49.2	48.8
PBC 1	51	49.4	48.2
PBC 2	51.2	50.6	49.6
PBC 3	50.8	49.8	50.1

**Table 2:** Wear rate and COF of pure PEEK specimen

**Normal Load (N)**	**Sliding Velocit y (rpm)**	**Sliding Distance (m)**	**Wear Rate (x 10-14 m2/N)**	**Frictional coefficient (µ)**	**Weight before Test (gm)**	**Weight After Test (gm)**	**Change in weight (gm)**
20	350	1000	145.63	0.38	3.0337	3.0309	0.0028
20	350	2000	147.78	0.36	2.823	2.8194	0.0036
20	350	3000	149.92	0.34	3.057	3.047	0.01
20	350	4000	152.74	0.31	3.1135	3.1021	0.0114
40	350	1000	149.84	0.45	3.0233	3.0195	0.0038
40	350	2000	152.06	0.42	3.2155	3.2111	0.0044
40	350	3000	152.98	0.38	3.2279	3.2223	0.0056
40	350	4000	153.15	0.33	2.7499	2.7369	0.013

**Table 3:** Wear rate and COF of PBC 1 specimen

**Normal Load (N)**	**Sliding Velocit y (rpm)**	**Sliding Distance (m)**	**Wear Rate (x 10-14 m2/N)**	**Frictional coefficient (µ)**	**Weight before Test (gm)**	**Weight After Test (gm)**	**Change in weight (gm)**
20	350	1000	135.436	0.3641	3.8233	3.8218	0.0015
20	350	2000	135.995	0.3276	3.4013	3.3998	0.0015
20	350	3000	138.566	0.2821	3.7535	3.7507	0.0028
20	350	4000	142.048	0.2639	3.4706	3.4668	0.0038
40	350	1000	139.351	0.4095	3.3594	3.3546	0.0048
40	350	2000	142.137	0.3822	3.6732	3.6681	0.0051
40	350	3000	142.891	0.3458	3.4791	3.473	0.006
40	350	4000	143.229	0.3094	3.4922	3.4823	0.0099

**Table 4:** Wear rate and COF of PBC 2 specimen

**Normal Load (N)**	**Sliding Velocit y (rpm)**	**Sliding Distance (m)**	**Wear Rate (x 10-14 m2/N)**	**Frictional coefficient (µ)**	**Weight before Test (gm)**	**Weight After Test (gm)**	**Change in weight (gm)**
20	350	1000	124.514	0.3264	2.8563	2.853	0.0033
20	350	2000	125.497	0.3048	3.0981	3.0935	0.0046
20	350	3000	127.472	0.2632	3.2796	3.2749	0.0047
20	350	4000	130.593	0.2394	3.1364	3.1321	0.0043
40	350	1000	128.113	0.3564	3.9343	3.9325	0.0018
40	350	2000	129.005	0.3323	3.3778	3.3757	0.0021
40	350	3000	130.298	0.3106	2.8946	2.8935	0.0011
40	350	4000	130.943	0.2991	3.4839	3.483	0.0009

**Table 5:** Wear rate and COF of PBC 3 specimen

**Normal Load (N)**	**Sliding Velocit y (rpm)**	**Sliding Distance (m)**	**Wear Rate (x 10-14 m2/N)**	**Frictional coefficient (µ)**	**Weight before Test (gm)**	**Weight After Test (gm)**	**Change in weight (gm)**
20	350	1000	131.067	0.3241	3.0341	3.0365	0.0024
20	350	2000	132.102	0.3196	2.9321	2.9349	0.0028
20	350	3000	135.128	0.2866	3.1405	3.1436	0.0031
20	350	4000	137.466	0.2694	3.0027	3.0064	0.0037
40	350	1000	134.856	0.3771	3.2143	3.2178	0.0035
40	350	2000	135.584	0.3612	3.3892	3.3931	0.0039
40	350	3000	136.882	0.3368	3.2785	3.2831	0.0046
40	350	4000	137.835	0.3024	3.1736	3.1793	0.0057
